# Meta-Atoms with Toroidal Topology for Strongly Resonant Responses

**DOI:** 10.3390/mi14020468

**Published:** 2023-02-17

**Authors:** Odysseas Tsilipakos, Zacharias Viskadourakis, Anna C. Tasolamprou, Dimitrios C. Zografopoulos, Maria Kafesaki, George Kenanakis, Eleftherios N. Economou

**Affiliations:** 1 Theoretical and Physical Chemistry Institute, National Hellenic Research Foundation, GR-11635 Athens, Greece; 2 Institute of Electronic Structure and Laser, Foundation for Research and Technology-Hellas, GR-70013 Heraklion, Crete, Greece; 3 Section of Electronic Physics and Systems, Department of Physics, National and Kapodistrian University of Athens, GR-15784 Athens, Greece; 4 Consiglio Nazionale delle Ricerche, Istituto per la Microelettronica e Microsistemi (CNR-IMM), 00133 Rome, Italy; 5 Department of Materials Science Technology, University of Crete, GR-70013 Heraklion, Crete, Greece; 6 Department of Physics, University of Crete, GR-70013 Heraklion, Crete, Greece

**Keywords:** toroidal dipole, metasurfaces, multipole expansion, broken symmetry, 3D printing, microwaves

## Abstract

A conductive meta-atom of toroidal topology is studied both theoretically and experimentally, demonstrating a sharp and highly controllable resonant response. Simulations are performed both for a free-space periodic metasurface and a pair of meta-atoms inserted within a rectangular metallic waveguide. A quasi-dark state with controllable radiative coupling is supported, allowing to tune the linewidth (quality factor) and lineshape of the supported resonance via the appropriate geometric parameters. By conducting a rigorous multipole analysis, we find that despite the strong toroidal dipole moment, it is the residual electric dipole moment that dictates the electromagnetic response. Subsequently, the structure is fabricated with 3D printing and coated with silver paste. Importantly, the structure is planar, consists of a single metallization layer and does not require a substrate when neighboring meta-atoms are touching, resulting in a practical, thin and potentially low-loss system. Measurements are performed in the 5 GHz regime with a vector network analyzer and a good agreement with simulations is demonstrated.

## 1. Introduction

Toroidal multipoles are a class of fundamental electromagnetic excitations that complement the more familiar electric and magnetic multipole families [1,2,3,4,5]. The toroidal dipole, the lowest order member of the toroidal family, first considered by Zel’dovich [6], originates from conduction or displacement currents circulating on a torus along the meridians, producing a closed loop of magnetic field circulation. Although it is distinctly different in its construction from the electric dipole (p, a simple separation of electric charges), it emits radiation with the same angular momentum and parity properties as the electric dipole. As a result, the two cannot be easily discerned by observing the far field radiation.

Recently, metamaterials have provided fertile ground for the observation of the toroidal dipole and higher order multipoles through the ability to judiciously shape the meta-atom/meta-molecule geometry in the unit cell. The first demonstration was published in 2010 by Kaelberer et al. [7]. Since then, a broad range of structures have been studied, based on both dielectric materials (displacement currents) [8,9,10,11,12] and metals (conduction currents) [13,14,15]. In particular, planar and, ideally, single-metallization-layer structures are favorable for easier fabrication [16,17,18,19,20]. An important point of attention is the possibility to attain a quasi-dark (almost non-radiating) state, which is termed a “dynamic anapole”, and results from the near-destructive interference of the toroidal dipole, T, with the electric dipole, p [21]. As perfect destructive interference is approached through p+ikT→0, one can obtain arbitrarily high radiation quality factors, $Q_{\mathrm{rad}}$ (provided that contribution from other multipoles is suppressed). The total quality factor, $Q_{\mathrm{tot}}$, can be very high as well, provided that a material system with low absorption is used [22]. This concept is related to the topics of trapped [23], broken-symmetry [24,25] and BIC (bound states in the continuum) [26] resonances. In such cases, sharply resonant responses and narrow spectral linewidths can be achieved, which are particularly useful for functionalities requiring enhanced local fields. Examples of such applications with metasurfaces supporting anapole states are, for instance, nonlinear effects [27,28,29], lasers [30] and (bio)sensing [31,32].

In recent years, a broad range of metasurfaces (single-layer metamaterial structures) have been proposed for demonstrating toroidal dipole- and anapole-based phenomena [33]. In most cases, the designs are based on a toroidal topology. Here, we adopt such a design aimed at supporting an anapole state [13]; it consists of a unit cell geometry meant to enhance the toroidal character, it is planar and it requires a single metallization layer that can be free-standing (without a substrate), which are important advantageous traits regarding fabrication and practical applications. We show that, indeed, a quasi-dark resonant state can be supported, which leads to an arbitrarily narrow linewidth, limited only by the resistive quality factor. Varying the appropriate geometric parameters, both the linewidth *and* lineshape of the resonance can be controlled, offering valuable degrees of freedom in shaping the spectral response. By performing a rigorous multipole expansion, we find that the toroidal dipole moment in the structure is strong. However, the corresponding far-field scattering is cancelled exactly by the magnetic quadrupole, $Q^{m}$. Thus, a quasi-dark resonance is achieved by controlling the residual electric dipole moment through asymmetry in the unit cell geometry (central vs. outer gaps) and not by satisfying the anapole condition. Subsequently, we experimentally verify our calculations through measurements within a rectangular metallic waveguide setup. More specifically, toroidal meta-atom samples, fabricated by a very low-cost technique based on 3D printing and subsequent metallization with a conductive paste, are loaded in the waveguide cross-section, and the S parameters (reflection and transmission coefficients) are measured with a vector network analyzer.

One goal of our work is to demonstrate a practical, easily realizable meta-atom geometry that provides freedom in shaping the electromagnetic response in terms of both linewidth and lineshape. Importantly, this study also clearly demonstrates that an interpretation suggested by the unit cell geometry (its topology) does not necessarily lead directly to the physical interpretation of the phenomenon taking place. Instead, in order to reveal the actual physical behavior, a careful analysis should be performed in all cases. Thus, our work can act as a reference for the mindful discussion of the physical mechanisms behind the resonant response of toroidal metamaterials (but not limited exclusively to this special class of structures). In this paper, the physical evidence is provided by the rigorous multipole expansion and is supported by the experimental verification of the spectral response.

## 2. Materials and Methods

### 2.1. Metasurface Full-Wave Simulations and Multipole Expansion

The metasurfaces studied in this work are composed of a unit cell made of conductive material. In order to study the effect of finite conductivity and increasing resistive loss, conductivities in the range of 10${}^{3}$–10${}^{7}$ S/m have been assumed. Full-wave simulations are conducted with the finite element method (FEM) using the commercial software COMSOL Multiphysics. They concern scattering and guided-wave simulations with a CW excitation and eigenvalue simulations without excitation.

**Figure 1:**
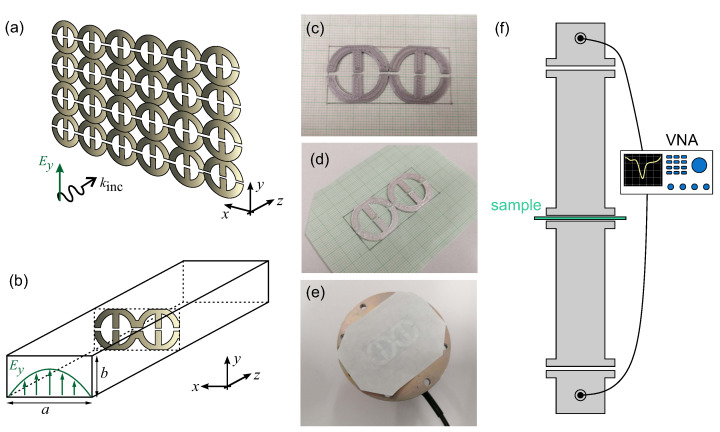
(**a**) Periodic metasurface illuminated with a normally incident plane wave of $E_{y}$ polarization. (**b**) Two unit cells fitted in a rectangular metallic waveguide. (**c**) 3D-printed unit cells using a PLA filament. (**d**) Structure after coating with a conductive silver paste. (**e**) Test fitting of the structure under study on the flange of the waveguide-to-coax adapter. The unit cells are lightweight and can be simply glued on a piece of paper and inserted at any junction between waveguide segments. (**f**) Measurement setup including a vector network analyzer allowing to measure reflection (S${}_{11}$) and transmission (S${}_{21}$) coefficients.

Two types of metasurfaces are considered. The first is a free-space metasurface, where the excitation is a normally incident plane wave with $E_{y}$ polarization (Figure 1a). In this case, a single unit cell is simulated with periodic boundary conditions in the *x*- and *y*-axes. In the second case, two periods of the metasurface along the *x*-axis are inserted within a rectangular metallic waveguide (Figure 1b), since the aspect ratio of the waveguide cross-section (a×b) is approximately 2:1. The structure is excited with the TE${}_{10}$ mode of the waveguide, exhibiting the characteristic field distribution $E_{y}\left(x\right)∼\sin\left(\pi x/a\right)$. The waveguide walls are modeled as perfect electric conducting (PEC) walls.

The multipole expansion is performed for the free-space geometry by using the expressions found in ref. [3]. The field scattered by the terms of the multipole expansion can be used for reconstructing the reflected and transmitted field [3,34,35]. The correctness of the calculated multipole moments is checked through the agreement between the direct and reconstructed reflection/transmission. This way, we can also specify where to truncate the multipole expansion without sacrificing reconstruction accuracy.

### 2.2. Meta-Atom Fabrication with 3D Printing

For a fast and cost-effective fabrication of the metasurface under study, 3D printing technology was employed. A commercial fused filament fabrication (FFF) 3D printer was utilized (MakerBot Replicator 2x, New York, NY, USA). A common polylactic acid (PLA) filament was used as a spool material for building the unit cell geometry (Figure 1c). Details regarding the printing process and conditions can be found in refs. [36,37]. Free-standing meta-atoms, such as those depicted in Figure 1c, were successfully fabricated. The fabricated metasurfaces exhibit negligible electrical conductivity, since PLA is an insulator. Therefore, the meta-atoms were subsequently coated with a thin (∼100 μm) layer of conductive silver paste, as shown in Figure 1d. The silver epoxy exhibits electrical conductivity as high as 10${}^{4}$–10${}^{5}$ S/m. We have deduced these values in earlier works [36,37] by comparing experimental results with simulations. Such a meta-atom configuration, in which an insulating core is coated with an adequately thick metallic paint, has been successfully employed at microwave frequencies [36,38].

### 2.3. Electromagnetic Characterization with Rectangular Waveguide Setup

The electromagnetic response of the investigated structure was experimentally confirmed through microwave measurements. To obtain a well-defined and highly reproducible measurement environment, which is not susceptible to external perturbations, we employed a rectangular metallic waveguide setup. More specifically, we fitted 2×1 unit cells inside the waveguide to fill the cross-section (aspect ratio 2:1). A test fitting of the two-unit-cell structure within the cross-section of a waveguide-to-coax adapter is shown in Figure 1e.

Measurements were conducted in the vicinity of 5 GHz by using an HP 8722ES vector network analyzer (Agilent Technologies Inc., Santa Clara, CA, USA). WR-187 rectangular waveguides (cross-section: 47.55 mm × 22.2 mm) were used, able to cover the frequency range of 3.95–5.85 GHz. The measurement setup is depicted in Figure 1f and allows for measuring both the reflection ($S_{11}$) and transmission ($S_{21}$) coefficients.

## 3. Results

### 3.1. Free-Space Metasurface with Controllable Strongly Resonant Response

**Figure 2:**
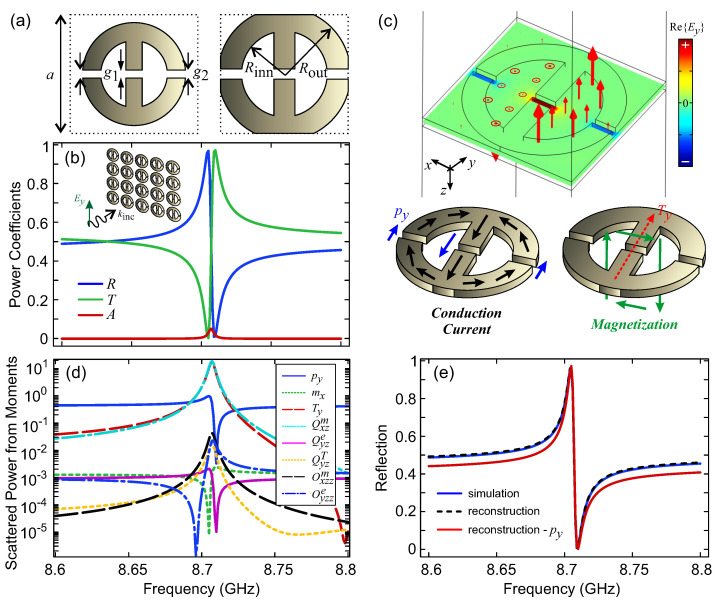
(**a**) Meta-atom geometry. When $R_{\mathrm{out}}>a/2$, the neighboring meta-atoms are touching; in this case, no substrate is necessary. (**b**) R/T/A power coefficients for plane-wave scattering by the periodic metasurface under normal incidence ($E_{y}$ polarization). The dimensions are a=15 mm, $R_{\mathrm{out}}=7$ mm, $R_{\mathrm{inn}}=5$ mm (w=2 mm), $g_{1}=0.5$ mm and $g_{2}=0.5$ mm. The thickness of the conductive meta-atom is h=1 mm. (**c**) Supported resonance associated with the spectral feature in panel (**b**). The color corresponds to the $E_{y}$ component (real part) and the arrows correspond to the magnetic field distribution. The eigenmode is characterized by a residual electric dipole moment due to counteracting contributions from the inner gap vs. the outer gaps. The magnetic field circulation gives rise to a strong toroidal dipole moment, $T_{y}$. (**d**) Power scattered from each multipole. The toroidal dipole and magnetic quadrupole moments are dominant at the resonant frequency. However, their scattered fields cancel out, since $E_{\mathrm{sca}}^{T_{y}}=-E_{\mathrm{sca}}^{Q_{xz}^{m}}$ exactly. Thus, the response is dictated by the residual electric dipole moment, $p_{y}$. (**e**) Comparison of the reflection coefficient calculated from the full-wave simulation with those reconstructed from the multipole moments. The response can be described fairly accurately when only the electric dipole moment is considered.

The meta-atom considered in this work is depicted in Figure 2a. Its geometry is selected in such a way so as to lead to a circulation of induced magnetization giving rise to a toroidal dipole moment [13]; we will come back to this while discussing Figure 2c. The gap in the inner branch is denoted by $g_{1}$ and the gaps in the outer branches are denoted by $g_{2}$. For the inner and outer radii, $R_{\mathrm{inn}}$ and $R_{\mathrm{out}}$, it holds $R_{\mathrm{inn}}=R_{\mathrm{out}}-w$, where *w* is the width of the central and outer branches. The lattice constant (pitch) is denoted by *a* for both *x* and *y* axes (square periodicity). When $R_{\mathrm{out}}>a/2$, the neighboring meta-atoms are touching; in this case, the conductive meta-atoms can form an interconnected metasurface and no substrate is strictly required. This can be important for avoiding additional resistive loss from the substrate and avoiding excess thickness. Preserving the vertical symmetry of the structure has been also shown to lead to higher modulation depth in transmission [39].

First, we examined a periodic metasurface. The reflection/transmission/absorption power coefficients (R/T/A) for plane wave scattering under normal incidence ($E_{y}$ polarization) are depicted in Figure 2b. The dimensions of the specific example are a=15 mm, $R_{\mathrm{out}}=7$ mm, $R_{\mathrm{inn}}=5$ mm (w=2 mm), $g_{1}=0.5$ mm and $g_{2}=0.5$ mm. The height of the conductive meta-atom is h=1 mm. In this case, the meta-atoms are not touching. We observed a Fano lineshape associated with the excitation of a resonance at ∼8.71 GHz and its interference with a non-resonant electric polarizability background. Even if the gaps are closed and the meta-atom is non-resonant, the conductive unit cell would still produce significant reflection. The conductivity adopted in these simulations is high ($\sigma =10^{7}$ S/m), leading to low absorption.

We subsequently performed an eigenvalue analysis to identify the nature of the supported resonance. The mode profile is depicted in Figure 2c; the color corresponds to the $E_{y}$ component (real part) and the arrows to the magnetic field. The magnetic field circles around the central branch and this should produce a toroidal dipole moment along the *y* axis ($T_{y}$). Looking at the fields in the gaps, one realizes that the central electric dipole moment is opposed by the two contributions of the outer gaps; when the opposing $p_{y}$ contributions compensate each other exactly, no coupling via the electric dipole moment is possible. This balance can be used to tune the radiative strength of an electrically coupled meta-atom [40].

To further study the physics of the scattering process, we performed a multipole expansion based on the induced conduction currents on the meta-atom using the expressions found in ref. [3], and we focused on the multipole moments that produce scattered fields toward the *z*-axis with $E_{y}$ ($H_{x}$) polarization. The eight leading terms of the expansion that satisfy the above criterion are $p_{y}$, $m_{x}$, $T_{y}$, $Q_{xz}^{m}$, $Q_{yz}^{e}$, $Q_{yz}^{T}$, $O_{xzz}^{m}$ and $O_{yzz}^{e}$. The results are depicted in Figure 2d. It can be seen that the rationale behind the meta-atom geometry has indeed resulted in a strong toroidal dipole moment contribution in the spectral neighborhood of the resonance frequency. However, the magnetic quadrupole moment is equally dominant. In fact, their scattered fields cancel out, since on resonance it holds exactly $E_{\mathrm{sca}}^{T_{y}}=-E_{\mathrm{sca}}^{Q_{xz}^{m}}$. As a result, the response is dictated by the residual electric dipole moment, $p_{y}$, which is the third most dominant contribution. This can be further verified in Figure 2e. We first confirm the validity of the multipole expansion by reconstructing the reflection coefficient. The agreement is excellent and shows that using the eight leading terms up to the electric octupole is more than adequate in this case. Importantly, if we use only the field scattered by the electric dipole moment, we obtain a fairly accurate description of the scattering process, especially near the resonance, where there is mutual cancellation of the dominant $T_{y}$ and $Q_{xz}^{m}$ terms.

**Figure 3:**
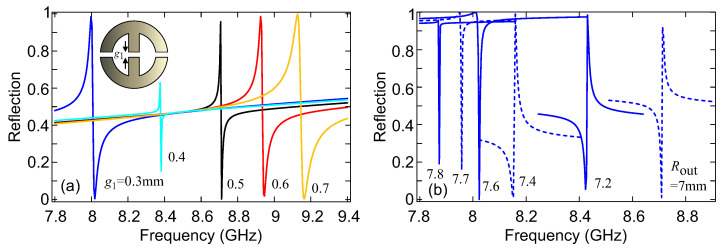
(**a**) Tuning the central gap ($g_{1}$), while keeping other gaps ($g_{2}$) constant at 0.5 mm. This controls the residual electric dipole moment and consequently the radiative strength of the resonance. (**b**) Varying the outer radius for $R_{\mathrm{inn}}=R_{\mathrm{out}}-w$ with w=2 mm. As the radius increases, the non-resonant (background) electric dipole moment changes and with it the Fano lineshape. For $R_{\mathrm{out}}$ values of 7.6, 7.7, and 7.8 mm, the meta-atoms are touching; this modifies the residual electric dipole moment, as the outer gaps of each meta-atom begin to merge with those of the neighboring one.

Next, we investigated different geometric degrees of freedom for tuning the resonance linewidth and lineshape. In Figure 3a, we varied the central gap ($g_{1}$) while keeping the outer gaps ($g_{2}$) constant at 0.5 mm. This controls the residual electric dipole moment and consequently the radiative strength of the resonance. For $g_{1}=0.4$ mm, the two opposing contributions (see Figure 2c) cancel each other out almost perfectly. In this case, very little coupling via the electric dipole moment is possible and the resonance becomes quasi-dark, resulting in a very narrow linewidth (high quality factor). The non-negligible reflection that remains is due to the non-resonant electric polarizability background. For $g_{1}>0.4$ mm, the central $p_{y}$ contribution dominates over those originating from the outer gaps, while the opposite occurs for $g_{1}<0.4$ mm. In both cases, the linewidth (and radiative strength) increases. The corresponding total quality factors are compiled in Table 1. They have been obtained from the solution of an eigenvalue problem using the complex eigenfrequency $\tilde{\omega }=\omega^{\prime }+i\omega^{\prime \prime }$ (the eigenvalue) through the expression $Q=\omega^{\prime }/\left(2\omega^{\prime \prime }\right)$. For additional details regarding approaches to calculating the quality factor we refer the interested reader to ref. [22]. We document the total quality factor, $Q_{\mathrm{tot}}$, which is directly associated with the linewidth of the spectral feature, along with the corresponding resonant frequency. Note that $Q_{\mathrm{rad}}\approx Q_{\mathrm{tot}}$ holds, since $Q_{\mathrm{res}}\to \infty$ (“res” stands for resistive) for such a high value of the conductivity ($\sigma =10^{7}$ S/m).

A different option is explored in Figure 3b, where we tune the outer radius. In all cases, the inner radius is also appropriately modified, so that the branch width is kept constant, i.e., $R_{\mathrm{inn}}=R_{\mathrm{out}}-w$, with w=2 mm. This primarily modifies the non-resonant (background) electric dipole moment, leading to changes in the Fano lineshape from highly asymmetric to quasi-Lorentzian. For $R_{\mathrm{out}}$ values of 7.6, 7.7, and 7.8 mm, the meta-atoms are touching, this additionally modifies the residual electric dipole moment, as the outer gaps of each meta-atom begin to merge with those of the neighboring atom, producing high values of background reflectance. Importantly, by tuning $R_{\mathrm{out}}$ we are able to achieve very narrow linewidths and, at the same time, a large resonance depth (compare Figure 3b with Figure 3a). The total quality factors for $R_{\mathrm{out}}=7.7$ and 7.8 mm are ∼5000 and ∼30,000, respectively. Note that this quality factor corresponds to radiation losses and will drop if resistive losses increase.

### 3.2. Meta-Atoms in a Rectangular Waveguide Setup—Experimental Verification

In this Section, the unit cells under study are inserted inside a rectangular waveguide environment (see Figure 1). This is exercised in order to end up with a well-defined and highly reproducible measurement environment, which is not susceptible to external perturbations. Furthermore, the electromagnetic response is anticipated to be similar to the free-space periodic structure, since the top/bottom PEC walls emulate a periodic repetition along the *y* axis and the main difference is the sin(πx/a) profile of the incident guided wave along the *x* axis [35,37,41].

**Figure 4:**
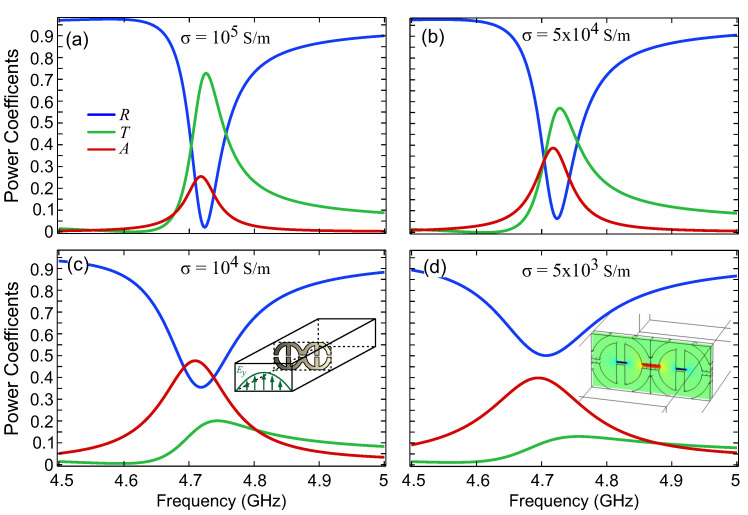
Simulations of the 3D-printed conductive unit cells within the WR-187 rectangular waveguide. The reflection ($R=|S_{11}{|}^{2}$), transmission ($T=|S_{21}{|}^{2}$) and absorption ($A=1-|S_{11}{|}^{2}-{\left|S_{21}\right|}^{2}$) power coefficients are plotted for different conductivities: (**a**) $\sigma =10^{5}$ S/m, (**b**) $\sigma =5\times 10^{4}$ S/m, (**c**) $\sigma =10^{4}$ S/m and (**d**) $\sigma =5\times 10^{3}$ S/m. As the conductivity is decreased, the spectral feature becomes broader and the absorption on resonance increases up to a maximum of 0.5 before starting to decrease again.

We first performed simulations. We tuned the dimensions in order to bring the resonant frequency near to the center of the band of the WR-187 rectangular waveguide (3.95–5.85 GHz) and fit 2×1 meta-atoms in the cross-section (a×b=47.55 mm × 22.2 mm). The dimensions are $R_{\mathrm{out}}=12$ mm, $R_{\mathrm{inn}}=8.6$ mm, w=3.4 mm, $g_{1}=0.7$ mm and $g_{2}=1$ mm. The thickness of the conductive meta-atom is h=3.4 mm. The response is depicted in Figure 4, where the reflection, $R=|S_{11}{|}^{2}$, transmission, $T=|S_{21}{|}^{2}$, and absorption, $A=1-|S_{11}{|}^{2}-{\left|S_{21}\right|}^{2}$, are plotted. The meta-atoms are touching and the resonance lineshape resembles the respective cases in Figure 3b. The meta-atoms will be fabricated via first 3D printing a dielectric (PLA) scaffold and then by coating with a silver paste, which is characterized by a limited conductivity of approximately σ = 10${}^{4}$–10${}^{5}$ S/m. Thus, in Figure 4 we examine the conductivities $\sigma =10^{5}$ S/m, $\sigma =5\times 10^{4}$ S/m, $\sigma =10^{4}$ S/m and $\sigma =5\times 10^{3}$ S/m. As the conductivity decreases, the spectral features become broader and the absorption increases up to a maximum of 0.5 (for an electrically polarizable structure) before starting to decrease again (under-coupling regime). The mode profile is depicted in the inset of Figure 4d. Notice that the gaps near the edges of the waveguide do not accommodate strong fields, since the incident TE${}_{01}$ waveguide mode is zeroed out at the side walls (cf. Figure 2).

**Figure 5:**
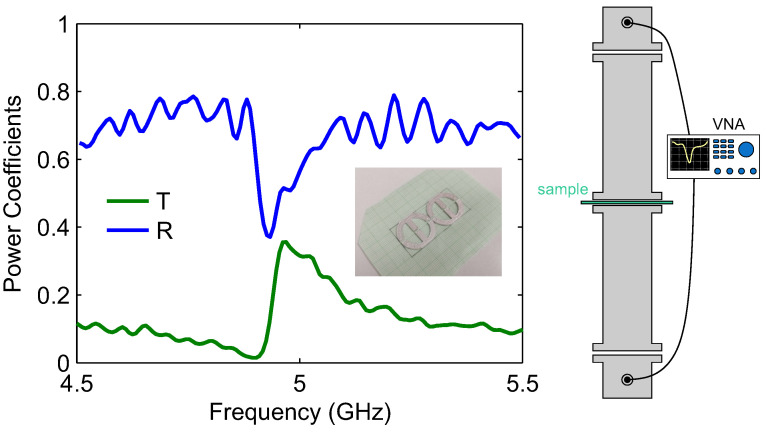
Measurement of the 3D-printed conductive unit cells within the WR-187 rectangular waveguide. The reflection ($R=|S_{11}{|}^{2}$) and transmission ($T=|S_{21}{|}^{2}$) power coefficients are plotted in the frequency range of 4.5–5.5 GHz.

In Figure 5, we depict the measurements of the 3D-printed conductive unit cells within the WR-187 rectangular waveguide. The reflection ($R=|S_{11}{|}^{2}$) and transmission ($T=|S_{21}{|}^{2}$) power coefficients are plotted in the frequency range of 4.5–5.5 GHz. The lineshape of the spectral feature and the linewidth (full-width half maximum of approximately 100 MHz, as extracted from the spectral feature in reflection) are in good qualitative agreement with those predicted by the simulations and specifically Figure 4c, which corresponds to a conductivity value we anticipate for the silver epoxy. The resonant frequency is slightly different; we attribute this discrepancy to the limited accuracy of the geometric dimensions achieved in the actual fabricated sample.

## 4. Discussion and Conclusions

We have studied a conductive meta-atom of toroidal topology, meant to enhance the toroidal dipole moment. We have performed simulations of both a periodic free-space metasurface comprised of this meta-atom and, subsequently, we have inserted two such meta-atoms into a metallic, rectangular waveguide setup. The latter configuration has allowed for experimentally verifying the resonance characteristics (lineshape and linewidth) and thus supports the physical claims arising from the theoretical analysis and multipole expansion.

One goal of our work was to demonstrate a practical, easily realizable meta-atom geometry that provides freedom in shaping the electromagnetic response in terms of both linewidth and lineshape. Another important goal was to reveal and highlight the physical mechanisms dictating the resonant response. Importantly, despite the physical interpretation suggested by the unit cell geometry (its toroidal topology), a rigorous multipole expansion has revealed that, although the toroidal dipole moment is indeed strong, the corresponding scattered field is cancelled out exactly by that of the magnetic quadrupole. As a result, the scattering response can be described accurately by simply considering the electric dipole moment alone. Thus, we conclude that a multipole expansion must be performed in all cases, in order to reach safe conclusions regarding the multipole components that mediate the scattering process.

The studied meta-atom allows to control the residual electric dipole moment through the geometric asymmetry between the central and outer gaps. This is a very similar concept to “accidental BIC” or “trapped/broken-symmetry” resonances. It becomes possible to achieve a controllably sharp resonant response, reaching very high radiation quality factors ($Q_{\mathrm{rad}}$). This is also possible in finite meta-atom arrays through spatially extended dark (sub-radiant) eigenmodes, as has been discussed in ref. [24]. Note, however, that ultimately the spectral linewidth is dictated by the total quality factor ($Q_{\mathrm{tot}}$), which will be limited by resistive losses as well. As a result, pushing for very high $Q_{\mathrm{rad}}$ values which require slight asymmetry and precise fabrication is not necessary when it cannot be supported by low material losses. Interconnected meta-atoms that do not require a substrate can help to avoid excess losses stemming from the substrate material and excess radiation losses stemming from the breaking of the vertical symmetry.

## Figures and Tables

**Table 1:** Quality factors for the cases depicted in Figure 3a. $g_{2}$ is held constant at 0.5 mm. They have been calculated using the complex eigenfrequency through $Q=\omega^{\prime }/\left(2\omega^{\prime \prime }\right)$.

Central Gap $g_{1}$ (mm)	Resonant Frequency (GHz)	$Q_{\mathrm{tot}}$
0.3	8.02	446
0.4	8.37	14,000
0.5	8.71	2,114
0.6	8.94	517
0.7	9.14	260

## Data Availability

The data presented in this study are available on request from the corresponding author.
